# Genetic, household and spatial clustering of leprosy on an island in Indonesia: a population-based study

**DOI:** 10.1186/1471-2350-6-40

**Published:** 2005-11-24

**Authors:** Mirjam I Bakker, Linda May, Mochammad Hatta, Agnes Kwenang, Paul R Klatser, Linda Oskam, Jeanine J Houwing-Duistermaat

**Affiliations:** 1 KIT (Koninklijk Instituut voor de Tropen/Royal Tropical Institute), KIT Biomedical Research, Meibergdreef 39, 1105 AZ Amsterdam, The Netherlands; 2 Department of Microbiology, Faculty of Medicine, Hasanuddin University, Kampus Tamalanrea KM 10, Jln. Perintis Kemerdekaan, 90245 Makassar, Indonesia; 3 Department of Medical Statistics and Bioinformatics, Leiden University Medical Centre, Postbus 9600, 2300 RC Leiden, The Netherlands

## Abstract

**Background:**

It is generally accepted that genetic factors play a role in susceptibility to both leprosy *per se *and leprosy type, but only few studies have tempted to quantify this. Estimating the contribution of genetic factors to clustering of leprosy within families is difficult since these persons often share the same environment. The first aim of this study was to test which correlation structure (genetic, household or spatial) gives the best explanation for the distribution of leprosy patients and seropositive persons and second to quantify the role of genetic factors in the occurrence of leprosy and seropositivity.

**Methods:**

The three correlation structures were proposed for population data (n = 560), collected on a geographically isolated island highly endemic for leprosy, to explain the distribution of leprosy *per se*, leprosy type and persons harbouring *Mycobacterium leprae*-specific antibodies. Heritability estimates and risk ratios for siblings were calculated to quantify the genetic effect. Leprosy was clinically diagnosed and specific anti-*M. leprae *antibodies were measured using ELISA.

**Results:**

For leprosy *per se *in the total population the genetic correlation structure fitted best. In the population with relative stable household status (persons under 21 years and above 39 years) all structures were significant. For multibacillary leprosy (MB) genetic factors seemed more important than for paucibacillary leprosy. Seropositivity could be explained best by the spatial model, but the genetic model was also significant. Heritability was 57% for leprosy *per se *and 31% for seropositivity.

**Conclusion:**

Genetic factors seem to play an important role in the clustering of patients with a more advanced form of leprosy, and they could explain more than half of the total phenotypic variance.

## Background

Leprosy, which annually affects about 700,000 new patients world wide [1], is a chronic disease caused by *Mycobacterium leprae*. It is thought that in endemic areas many individuals are infected with *M. leprae*, but only a few of those infected actually develop the disease [2]. Leprosy manifests itself as a disease spectrum, which for treatment purposes has been divided into two forms: multibacillary (MB) and paucibacillary (PB) leprosy. Clustering of leprosy patients within households, neighbourhoods and families has been reported several times [among others: [3-6]].

The discussion of the role played by genetic factors in the susceptibility to leprosy is long-standing, but came to prominence again with the recent identification of certain susceptibility loci [7-9]. In the past many studies have been performed on the genetic susceptibility of leprosy, such as segregation studies to unravel the mode of inheritance [10,11] and association studies to identify susceptibility genes [12,13]. Several reviews on this topic have been written [14,15]. It has been generally accepted that genetic factors do play a role in susceptibility to both leprosy *per se *and leprosy type and that probably multiple genes are involved. Suggestions have been made for a strong genetic component [16,17], but only few studies have tempted to quantify this. Wallace, Clayton and Fine [18] estimated the relative recurrence risk ratio to measure the genetic effect in Northern Malawi. They suggested that both genetic and non-genetic factors may play a role in the susceptibility to leprosy, with host genetics playing a small but significant role (siblings risk ratio was 2).

Interpretation of leprosy clustering within families or households is difficult, since those persons may not only share the same genes but also have close contact and the same or similar socio-economic circumstances. The main research question of this study was to test which correlation structure (genetic, household or spatial) gives the best explanation for the distribution of leprosy patients and persons harbouring specific anti-*M. leprae *antibodies (seropositive persons) in our study population. Furthermore, heritability estimates and the siblings recurrence risk ratio were calculated to give an indication of the maximum contribution of genetic factors to the occurrence of leprosy and seropositivity. The presence of *M. leprae *specific antibodies was used as a marker for leprosy infection, and was recently identified as a risk factor for MB leprosy [19]. The study was performed in a geographically isolated island population, that has a strong founder effect as it was founded by only a few persons about 100 years ago. Of this unique island population, which was found to be highly endemic for leprosy during a population survey in 2000 [6], the family structure was unravelled and an extended pedigree prepared.

## Methods

Prior to the study we received ethical clearance from the Ethical Research Committee of the Hasanuddin University and from the Ministry of Health of the Republic of Indonesia.

### Study population

The study described here was performed on the island Kembanglemari, which has 634 inhabitants and is situated in the Flores Sea. The island is part of Pangkep District of South Sulawesi Province in Indonesia and located 268 km from Makassar. The inhabitants originate from South Sulawesi and belong to the Mandar ethnic group.

### Data and sample collection

Clinical data were collected in June 2000. During an active door-to-door survey 88.3% of the population was examined for clinical symptoms of leprosy [6]. The diagnosis was based on the WHO classification. Patients with one lesion were classified as PB1 and with 2–5 lesions as PB2-5; patients with more than five lesions and/or with a positive bacterial index (BI) in at least one of three skin smears were classified as MB. Persons who reported to have completed a full course of multi-drug treatment, without active lesions and skin smear negative, were marked as patients released from treatment (RFT).

At the same time blood samples were collected of the population above 5 years: 68.1% of the population. Serum was separated by centrifugation on the same day and kept frozen until use.

During two subsequent population surveys in April 2002 and April 2003 the parent names of the majority of the inhabitants were asked. Furthermore, during the survey in April 2002 interviews were held with elderly people and leprosy patients about their family structure and ancestors. With these data an extended pedigree was prepared. To determine the occurrence of inbreeding the kinship coefficient (the probability that two alleles, at a randomly chosen locus, one chosen randomly from individual i and one from j are identical by descent) was computed for parents [20].

The longitudes and latitudes of every fifth house were measured using a hand-held Global Positioning System (GPS, Garmin, Kansas USA). In Arcview 3.2 (Esri, California USA) the remaining houses were situated between the geo-referenced houses using a detailed hand-drawn map. The resulting map was used to prepare a geographical distance matrix between all inhabitants.

### IgM antibody detection

The presence of IgM antibodies to *M. leprae *phenolic glycolypid I (PGL-I) was measured by an enzyme-linked immunosorbent assay (ELISA) as described previously [21] using the natural trisaccharide moiety of PGL-I linked to bovine serum albumin (NT-P-BSA) as antigen. Serum was diluted 1:500 and tested *in duplo*. The optical density at 450 nm (OD) of each serum sample was calculated by subtracting the OD value of BSA coated wells from that of NT-P-BSA coated wells. A positive reference serum on each plate was used to minimize plate-to-plate variation. The cut-off value for seropositivity was set at 0.200 [21]; any criterion for setting a cut-off is arbitrary since the distribution of antibody concentration is unimodal [22].

### Statistical analysis

Leprosy prevalence was defined as the proportion of the sum of leprosy patients and RFT patients over the population screened for leprosy in June 2000. Even though it is not common practice, for the purpose of this particular research question RFT patients were included in the prevalence. Seroprevalence was defined as the proportion of seropositive persons (including seropositive patients) over the population screened for antibodies.

A score statistic Q [23]

Q=∑i,j(zi−πi)Rij(zj−πj)∑i(zi−πi)2
 MathType@MTEF@5@5@+=feaafiart1ev1aaatCvAUfKttLearuWrP9MDH5MBPbIqV92AaeXatLxBI9gBaebbnrfifHhDYfgasaacH8akY=wiFfYdH8Gipec8Eeeu0xXdbba9frFj0=OqFfea0dXdd9vqai=hGuQ8kuc9pgc9s8qqaq=dirpe0xb9q8qiLsFr0=vr0=vr0dc8meaabaqaciGacaGaaeqabaqabeGadaaakeaacqWGrbqucqGH9aqpdaWcaaqaamaaqafabaGaeiikaGIaemOEaO3aaSbaaSqaaiabdMgaPbqabaGccqGHsisliiaacqWFapaCdaWgaaWcbaGaemyAaKgabeaakiabcMcaPiabdkfasnaaBaaaleaacqWGPbqAcqWGQbGAaeqaaOGaeiikaGIaemOEaO3aaSbaaSqaaiabdQgaQbqabaGccqGHsislcqWFapaCdaWgaaWcbaGaemOAaOgabeaakiabcMcaPaWcbaGaemyAaKMaeiilaWIaemOAaOgabeqdcqGHris5aaGcbaWaaabuaeaacqGGOaakcqWG6bGEdaWgaaWcbaGaemyAaKgabeaakiabgkHiTiab=b8aWnaaBaaaleaacqWGPbqAaeqaaOGaeiykaKYaaWbaaSqabeaacqaIYaGmaaaabaGaemyAaKgabeqdcqGHris5aaaaaaa@5851@

was used to test clustering of leprosy *per se*, MB leprosy, PB leprosy and seropositivity due to genetic, household and spatial effects. Here z_i _is the outcome for subject i (1 if affected and 0 otherwise), π_i _is the age and sex specific prevalence and R_ij _is the genetic, household or spatial correlation for subject i and j. The specific R_ij _are described below. In the simple case of π_i _= 0.5 for all i, the statistic Q reduces to the sum over concordant pairs (i,j) (for example leprosy patient - leprosy patient) of R_ij _minus the sum over disconcordant pairs (i,j) (leprosy patient - person with no leprosy) of R_ij_. In general the statistic Q tends to be large when concordant pairs have higher correlations R_ij _compared to discordant pairs. For the score test it is important to realise that healthy persons also provide information, although not as much as the patients. The distribution of Q under the null hypothesis of no correlation can be approximated by a chi-square distribution with scale parameter 0.5Var(Q)/E(Q) and degrees of freedom of 2E(Q)^2^/Var(Q). Formulae for the expectation and variance of Q can be found in Houwing-Duistermaat et al [23].

The correlation structures R_ij _corresponding to the genetic, household and spatial effects were based on distances between individuals. For the genetic model correlation between pairs is based on genetic distances (d^g^) in the pedigree; siblings have a higher correlation compared to cousin-pairs and unrelated persons have no correlation. Specifically R_ij _= 1/2^dg ^which is equivalent to two times the kinship coefficient. In the household model the distances between individuals sharing the same household is zero which gives a correlation of 1, and distance infinite for inhabitants of different households (R_ij _= 0). The spatial model is an extension of the household model. The distance for the spatial model (d^e^) equals the distance between 2 households in metres. We used the following formula: R_ij _= exp(-d^e^_ij_/44). In previous studies it was shown that apart from household contacts also first and second neighbours have an increased risk of developing leprosy [5]. The number 44 still gives a good correlation between a house and its second neighbour: for d^e ^= 11 (the mean distance between a house and its nearest (first) neighbour) R_ij _= 0.779, for d^e ^= 22 (the assumed distance between house and its second neighbour) R_ij _= 0.607 and for d^e ^= 33 R_ij _= 0.473. This last correlation is seen as a moderate correlation [24]. Thus, the correlation decreases when the distance between 2 households becomes larger. We performed a sensitivity analysis in which the number 44 was changed into 33 and 55. Spearman rank correlation coefficients were computed between the correlations R_ij _of the different random effects.

In the analysis for leprosy *per se *all patients detected in June 2000 and the RFT patients were included. These RFT patients were excluded from the separate analyses for MB and PB leprosy, because classification could not be confirmed. For leprosy *per se *the test was, apart from the total population, also performed on a subpopulation which was expected to have a relatively stable household status over the last 20 years, namely the population below 21 and above 39 years. From the data it was seen that up to the age of 20 84% (291/346) still lived in the same house as their mother, and that after the age of 20 this percentage was much smaller (12%; 25/214), indicating that most people moved when they were around 20 year of age. Interviews learned that most people move only once in their life, namely when they get married and move from their parental house into their own house. Persons aged 21–39 were excluded because it is expected that most of these persons had a change in household status within the last 20 years.

Heritability estimates were calculated for leprosy *per se *and for seropositivity using a random effects model with a logit link and assuming Gaussian random effects [25]. The heritability estimates are presented with two-sided 95% confidence intervals (95% CI). The confidence intervals were estimated using profile likelihood. Both the score statistic and the heritability estimates were adjusted for the covariates age (continuous) and sex.

Finally, the risk ratios for siblings (λs) for leprosy *per se *and seropositivity, defined as the ratio of the risk of leprosy/seropositivity for siblings of affected persons to the risk for the general population, were calculated separately for the group under 21 years and the group of 21 years and older according to the method described by Olson and Cordell [26]. Confidence intervals were calculated according to the method of Zou and Zhao [27]. Different age groups were used because the risk of leproys/seropositivity for the general population (i.e. prevalence) differed between age groups.

## Results

**Figure 1:**
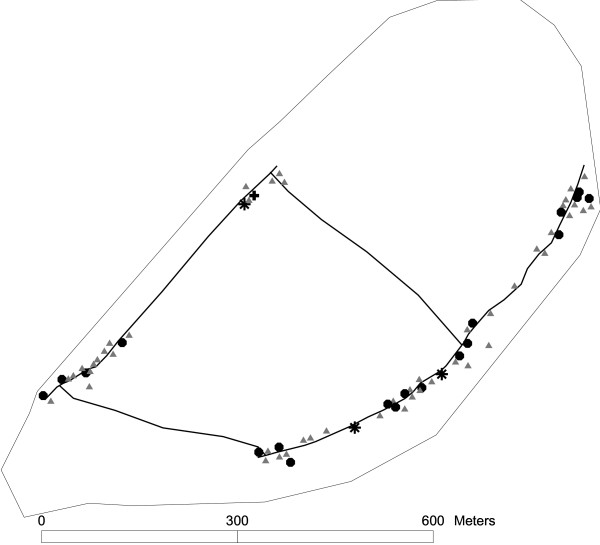
**Map of Kembanglemari Island showing the new patients per house**.  = 0, ● = 1,  = 2, + = 3 new patients. Not all houses without patients are shown.

**Figure 2:**
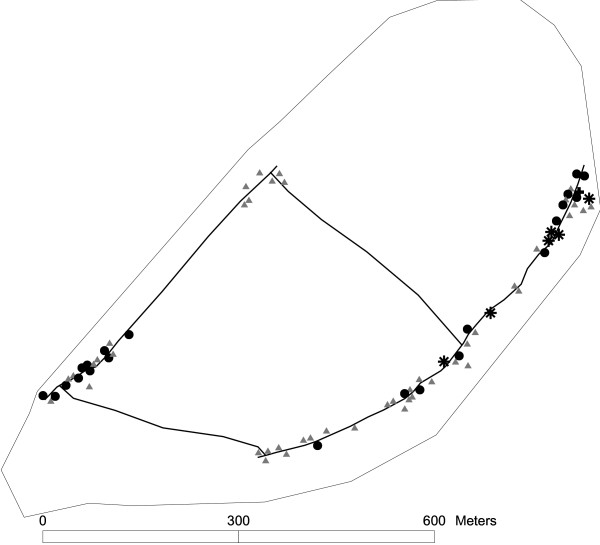
**Map of Kembanglemari Island showing the seropositive persons per house**.  = 0, ● = 1,  = 2, + = 3 seropositive persons. Not all houses without seropositive persons are shown.

In June 2000 634 persons were living in the 120 houses on Kembanglemari (average: 5.3 persons per house). Of the 560 persons screened, 28 were diagnosed with leprosy (12 were classified as MB, 3 as PB2-5 and 13 as PB1) and 3 persons were identified who had leprosy in the past, but were released from treatment (Table 1). Of the 432 persons who were tested for antibodies against *M. leprae*, 37 (8.6%) were seropositive. Two (17%) of the 12 MB patients, none of the PB patients and none of the RFT patients were seropositive. Table 2 shows leprosy and seroprevalence for the different age groups: leprosy was more prevalent among adults than among children while seroprevalence was higher among children. No difference was found between men and women with regard to leprosy (4.9% and 6.1%, respectively, p = 0.52) or seropositivity (7.4% and 9.6%, respectively, p = 0.41). Figures 1 and 2 show maps of the island with the patients and the seropositive persons per house, respectively.

**Figure 3:**
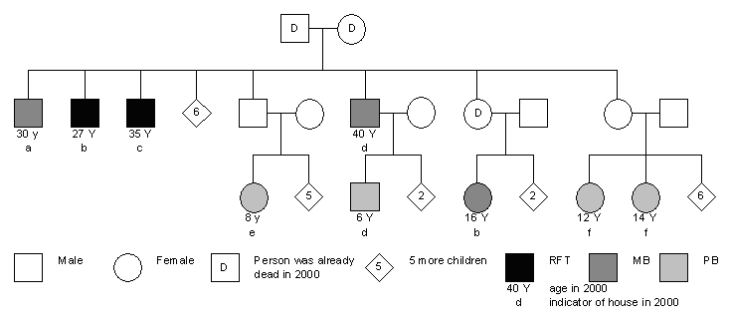
Part of the extended pedigree of family one, showing family relations between leprosy patients.

Four families were identified on the island. The pedigrees included 791 persons in total, of which 589 were living on the island in June 2000 and 202 persons were parents who had already died, lived somewhere else or could not be traced. One pedigree included 568 (89.6%) inhabitants, all leprosy patients and 35 seropositive persons and consisted of six generations. Figure 3 shows part of this pedigree as an example. Of the 560 persons screened for leprosy 535 were included in the pedigrees and of the 432 persons who were tested for antibodies 411 were included. The unrelated single individuals (25 in leprosy analyses and 21 in seropositivity analyses) were retained in the analysis as independents.

In total 184 couples with children were counted. Of 79 pairs who were alive in 2000, both parents were known. Of these, 15 pairs (19%) were genetically related (kinship coefficient varied between 0.156–0.0156). They had 62 children (56 screened for leprosy, 34 tested for antibodies). None of the children had leprosy and 4 were seropositive (11.8%).

For the total population the test statistic of Houwing-Duistermaat et al. [23] (results in Table 3) showed that for leprosy *per se *and MB leprosy the genetic correlation structure fitted best (p < 0.001 and p = 0.002, respectively). For MB leprosy the household model also fitted (p = 0.005). For PB leprosy neither the genetic nor the household nor the spatial correlation structure were significant. For seropositivity the spatial correlation structure fitted best (p = 0.003), but the genetic model was significant too (p = 0.016). For leprosy *per se *in the population below 21 and above 39 years the genetic correlation structure fitted best, but all structures were significant (p < 0.001 for genetic, p = 0.002 for household and p = 0.017 for spatial). The sensitivity analysis showed no substantial differences in the results when using 33 or 55 instead of 44 in the spatial correlation structure. In the total population, the Spearman rank correlation coefficient between the spatial and genetic correlations was 0.10, between the household and genetic correlations 0.15, and between the spatial and household correlations 0.16.

The heritability estimate for leprosy was 0.57 (95% CI: 0.22–0.81) and for seropositivity 0.31 (95% CI: 0–0.71). The risk ratio for siblings (λ_S_) for leprosy *per se *in the population below 21 years was 6.4 (95% CI: 0–17.0) and in the population above 20 years 2.9 (95% CI: 0–9.3). For seropositivity λ_S _was 1.3 (95% CI: 0–3.0) in the population <21 years and 2.7 (95% CI: 0–7.0) in the population >20 years.

## Discussion

### Strengths of the study

This study describes a unique island population with a strong founder effect: 90% of the population belonged to the same pedigree in which consanguineous marriages took place and the leprosy prevalence was extremely high (5.5%). This makes the population very suitable to study whether genetic effects can explain the distribution of leprosy-related traits within the families.

### Principal findings

In the total population clustering of leprosy *per se *could only be explained by genetic factors and not by contact status. In this particular population the heritability of leprosy *per se *was 57%. For PB leprosy no clustering could be detected, but for MB leprosy both the genetic and the household were significant. For seropositivity genetic factors seemed less important compared to leprosy: the heritability of seropositivity was lower, namely 31%, and although the genetic model was significant, the spatial model explained the clustering of seropositivity better.

### Potential biases

This population-based study included 560 persons of which 31 were affected with leprosy. Although the total population is large, and has one of the highest prevalences of leprosy in the world, the number of leprosy patients is still rather small. Therefore the confidence intervals of the heritability and siblings risk ratios are rather large, which may limit the weight that should be given to the results.

Since the three effects give similar correlation structures in the data (family members are for example living in the same house), a significant effect may be a confounder for one of the other effects [28]. Therefore the heritability estimates may also partly reflect shared environmental effects. Fortunately the correlations between the structures appeared to be rather small (≤0.16) indicating only small overlap between the various effects. The score statistics indicate which correlation structure fits best to the data. Thus the heritability estimate of 0.57 for leprosy represents genetic effects while the heritability of seropositivy probably also measures spatial effects, since the spatial correlation structure fitted better to the data than the genetic correlation structure.

Information bias could have occurred since leprosy patients were interviewed as a separate group and they may have had a better insight into their genetic distance to other leprosy patients. However, since information was collected in multiple ways, i.e. also by interviewing elderly persons and through two population surveys, it is expected that this bias will be minimal.

Household status as well as the distance matrix (used for the spatial model) were determined at the time of the screening in June 2000. The household status in 2000 does not necessarily reflect the household status at time of transmission/infection due to the long incubation time (estimated to vary between 2 and 12 years [29]). During this time patients could have moved to different houses. To overcome this problem we decided to apply the test statistic for leprosy *per se *also for the population excluding those aged between 20 and 40 years. Young adults seem to move out of their parental house around their 20^th ^birthday. The 20-year lag time is to take into account the incubation period and detection delay. We assumed that within the group younger than 20 and older than 40 years the household status for most patients would be similar at time of infection and diagnosis.

### Interpretation

In contrast to the results of the general population, where susceptibility to leprosy *per se *could only be significantly explained by genetic factors, among the population below 21 and above 39 years all three correlation structures were significant. Especially the household and genetic effects were highly significant in this subgroup, making it more difficult to distinguish between the effects. Genetically closely related persons probably live in the same household for this age group. The smallest p-value for genetic effects suggests, however, that genetically related individuals living in different households tend to have similar outcomes and/or distantly related individuals living in the same household have different outcomes.

When looking separately for MB and PB leprosy in the total population, MB leprosy appeared to have a genetic as well as a household effect. However, for PB leprosy clustering could not be detected at all, meaning that it was randomly spread in the population. The PB group consisted mainly of PB patients with a single lesion (78%). Part of these PB1 patients may have spontaneously healed if there had been no active screening [16]. The fact that we do not have many PB patients with 2 to 5 lesions, could be due to over-diagnosis of MB patients. In that way it would be better to describe the MB patients as the patients with a more advanced form of leprosy. It seems that for actual progression to a more advanced state of disease genetic factors become more important.

Although not significant, probably due to small numbers, a relatively high recurrence risk ratio of 6.4 for siblings for leprosy *per se *was found in the population below 21 years, indicating that brothers/sisters of a patient had a more than 6 times higher risk of developing leprosy compared to the general population in that age group. Since in this young population most of the siblings still live together in the parental house, the recurrence risk ratio measures also household effects. In the population above 20 years the recurrence risk ratio for siblings was 2.9, which is comparable to other studies: in a population in South India a λ_s _of 2.4 for tuberculoid leprosy [30] and in a population in Vietnam a λ_s _of 2.21 for leprosy *per se *[17] has been estimated. The λ_s _for infectious diseases usually lies between 1.5–5 which is much lower than for example for autoimmune diseases like type 1 diabetes and multiple sclerosis with λ_s _between 15–20 [31].

The recurrence risk ratio for siblings measures the excess risk of siblings of affected persons compared to the population risk and could be used for developing rational screening procedures. However, it is not adjusted for age or sex and only uses the information of clustering within siblings. Moreover within siblings the effect of sharing households will be relatively large. In contrast, the heritability estimates are based on correlation between all genetically related subjects and thus also on relatives who do not share household for example cousin pairs. It estimates the proportion of the genetic variance explaining the total phenotypic variance in a defined population and is used to quantify the degree of genetic contribution to the development of a disease. We found that in the total population for leprosy *per se *57% of the total variance could be explained by genetic factors. Probably MB leprosy is responsible for most of this, since the genetic effect was highly significant for MB leprosy but not significant for PB leprosy. Only one other study, performed in the Philippines, described a heritability of lepromatous leprosy among men of 80% [32]. However, this relatively high estimate is only based on siblings and thus the contribution of shared environment to the estimate may be relatively high.

The distribution of seropositive persons could be explained best by the spatial model, but the genetic model was also significant. The household model could not significantly explain clustering. A heritability of 31% was found and in the population below 21 years siblings of seropositive persons did not have an increased risk to be seropositive compared to the general young population. In the population above 20 years the recurrence risk ratio for siblings was 2.7, which is comparable with that for leprosy *per se*. It seems that especially in the young population genetic factors are less important for seropositivity than for leprosy, which could be reflected by the high seroprevalence in the young population. In a recent publication we showed that living in the vicinity of two seropositive patients increased the risk of harbouring antibodies against *M. leprae *[33]. It seems that having contact with an infectious patient is an important factor in harbouring antibodies, but to develop MB leprosy genetic factors become more important.

Leprosy and seroprevalence were not significantly increased among children of genetically related parents. However, the sample size was rather small (56 and 34 children, respectively), which makes it difficult to draw conclusions. In our study genetically related parents have a higher chance to appear in the younger generations of the pedigree, since in the older generations the correlation between parents was often unknown. The fact that we found 15 pairs of related parents, of which three persons who married a related person also had parents who were related, indicates that marrying a related person is customary on this island. This suggests that also in the older generations of the family tree inbreeding may have occurred, which makes this population interesting for studying recessive genes.

### Use of study population for detection of genes

Until now genome scans that are published either studied the phenotypes leprosy *per se *[17] or PB leprosy [7]. Here we showed that in our population genetic factors appear to be important for advanced forms of leprosy. A logical next step would be to see which (candidate) genes, such as the PARK2 and PACRG genes [9], could explain the genetic effect found in this population. If none of the already known chromosome regions can explain the effect, a genome-wide scan could be carried out to detect new regions.

## Conclusion

Since leprosy is thought to spread from person to person, contact with a leprosy patient is essential for the transmission of *M. leprae*. Among many factors that could influence the development of infection and disease, such as age, nutritional status and contact with other mycobacteria, genetic factors probably also play a role. In this highly endemic area for leprosy genetic factors could explain up to 57% of the total variance. This unique study population is very suitable to confirm the role of already known chromosome regions in controlling leprosy or to search for new susceptibility loci.

## Competing interests

The author(s) declare that they have no competing interests.

## Authors' contributions

MB was involved in study design, data collection and statistical analyses and finalized the manuscript. LM was involved in study design, data collection and wrote the first draft of the paper. MH was involved in study design and coordinated the data collection. AK participated in implementation and data collection. PK was involved in study design and interpretation of results. LO was involved in study design and supervision. JH performed the statistical analysis and interpreted results. All authors read and approved the final manuscript.

## Pre-publication history

The pre-publication history for this paper can be accessed here:



## Figures and Tables

**Table 1:** Characteristics of study population in June 2000

	n	%
Persons living on Kembanglemari	634	
Persons screened for leprosy	560	88.3
Persons screened for antibodies	432	68.1
Persons included in pedigrees	589	92.9
		
Leprosy status		
No leprosy	529	94.5
PB1^a ^leprosy	13	2.3
PB2-5^b ^leprosy	3	0.5
MB^c ^leprosy	12	1.9
RFT^d ^(no classification available)	3	0.5
		
Serological status		
Seronegative	395	91.4
Seropositive	37	8.6

^a ^PB1 = single lesion paucibacillary leprosy

^b ^PB2-5 = paucibacillary leprosy with 2–5 lesions

^c ^MB = multibacillary leprosy

^d ^RFT = released from treatment

**Table 2:** Leprosy and seroprevalence per agegroup

Age group (years)	Screened for leprosy	RFT^a^	MB Leprosy^b^	PB Leprosy^c^	Leprosy N (%)	Tested for IgM	Seropositive N (%)
0–5	122	0	0	0	0	7	1 (14.3)
6–20	219	0	4	8	12 (5.5)	211	24 (11.4)
21–39	136	2	5	5	12 (8.8)	133	9 (6.8)
40–59	57	1	3	0	4 (7.0)	55	1 (1.8)
≥60	26	0	0	3	3 (11.5)	26	2 (7.7)

Total	560	3	12	16	31 (5.5)	432	37 (8.6)

^a ^RFT = released from treatment

^b ^MB = multibacillary leprosy

^c ^PB = paucibacillary leprosy

**Table 3:** P-values for testing the null hypothesis of no clustering of leprosy *per se*, MB leprosy, PB leprosy and of seropositivity due to genetic, household and spatial effects.

**Study population **Trait	N	(sero)prev^e^.	Genetic	Household	Spatial
**Total population**					
Leprosy *per se*	560	5.5%	***<0.001***	0.084	0.145
MB^a ^leprosy	541^c^	2.2%	***0.002***	*0.005*	0.105
PB^b ^leprosy	545^d^	2.9%	0.311	0.154	0.555
Seropositivity	432	8.6%	*0.016*	0.118	***0.003***
**Population <21 and >39 years**					
Leprosy *per se*	424	4.5%	***<0.001***	*0.002*	*0.017*

Test-statistic is adjusted for age and sex.

*Italic*: p-value <0.05; **Bold**: best fitting model

^a ^MB = multibacillary leprosy; ^b ^PB = paucibacillary leprosy

^c ^541 = 560 - 3 RFT patients - 16 PB patients

^d ^545 = 560 - 3 RFT patients - 12 MB patients

^e ^(sero)prev = leprosy prevalence or seroprevalence
